# Mean arterial pressure is associated with the neurological function in patients who survived after cardiopulmonary resuscitation: A retrospective cohort study

**DOI:** 10.1002/clc.23502

**Published:** 2020-11-18

**Authors:** Hai‐bo Ai, Hong Zhang

**Affiliations:** ^1^ Rehabilitation Medicine Department, The Third Hospital of Mianyang, Sichuan Mental Health Center Mianyang Sichuan China

Thank you for Kosuke Miki's letter. We fully agree that the measurement time of the mean arterial pressure (MAP) is an essential part of our study, especially for the clinical application of the study. However, due to our carelessness, our study did not clearly express the measurement time of the MAP, which confused readers. In our study, MAP refers to the MAP measured during ICU (intensive care unit) admission. As for the effect of blood pressure raising methods and anticoagulant therapy mentioned by the author on the neurological function of patients after CPR (cardio‐pulmonary resuscitation), our study cannot solve this problem, and more studies may be needed.

## Data Availability

NA

